# Human brain and organoid transcriptomes reveal key receptor tyrosine kinase pathways and genetic signatures in Alzheimer's disease

**DOI:** 10.1038/s12276-026-01684-5

**Published:** 2026-04-15

**Authors:** Saewoon Shin, Xiaohui Zhu, Sarnai Amartumur, Taehoon Lee, Won Jong Yu, Soomin Park, Niloofar Etemadi, Ariunzaya Jamsranjav, Rian Kang, Gyusoo Bak, Dongjoon Lee, Jieun Kim, Jong Won Han, Chaejeong Heo, Hansang Cho, Sunghoe Chang, Inhee Mook-Jung, Sang-Eun Lee, Jong-Chan Park

**Affiliations:** 1 Department of Biophysics, Sungkyunkwan University, Gyeonggi-do, Republic of Korea; 2 Institute of Quantum Biophysics, Sungkyunkwan University, Gyeonggi-do, Republic of Korea; 3 Graduate School of Stem Cell and Regenerative Biology, Korea Advanced Institute of Science and Technology (KAIST), Daejeon, Republic of Korea; 4 Department of Biopharmaceutical Convergence, Sungkyunkwan University, Suwon, Republic of Korea; 5 Department of Metabiohealth, Sungkyunkwan University, Gyeonggi-do, Republic of Korea; 6 Department of Biochemistry and Biomedical Sciences, College of Medicine, Seoul National University, Seoul, Republic of Korea; 7 Department of Physiology and Biomedical Sciences, College of Medicine, Seoul National University, Seoul, Republic of Korea; 8 Neuroscience Research Institute, Seoul National University Medical Research Center, Seoul, Republic of Korea; 9 Convergence Dementia Research Center, College of Medicine, Seoul National University, Seoul, Republic of Korea; 10 Department of Life Sciences, CHA University, Seongnam, Gyeonggi Republic of Korea

**Keywords:** Alzheimer's disease, Molecular neuroscience

## Abstract

Alzheimer disease (AD) is a progressive neurodegenerative disorder marked by transcriptomic alterations affecting multiple genes. Many researchers have tried to predict major hallmarks of AD pathogenesis for diagnosis but the association between receptor tyrosine kinase (RTK) pathways and AD diagnosis is still unclear. This study aims to identify RTK-associated gene signatures crucial to AD pathogenesis and assess their potential as diagnostic biomarkers for AD. The study investigated changes in RTK pathway gene expression related to AD by analyzing brain transcriptome data from two independent public data sets (GSE84422 and GSE109887). Differentially expressed genes (DEGs) were analyzed from the GSE84422 and GSE109887 data sets and overlapping genes (oDEGs) were identified. RTK-related genes (ooDEGs) were subsequently selected through functional enrichment analysis. These were further refined into AD-related genes (disease-associated genes (DAGs)) through protein–protein interaction network analysis. Logistic regression and receiver operating characteristic analyses were conducted on the selected DAGs to evaluate their diagnostic potential, with additional gene expression validation performed in brain organoids and primary neurons. A total of 145 genes were identified as oDEGs in the above two data sets, and 18 genes were selected as ooDEGs. Six DAGs (*ITGB1*, *AXL*, *GFAP*, *NRG1*, *CAV1*, and *RHOA*) were selected. The diagnostic powers of the six DAGs for AD were 0.825 (GSE84422) and 0.884 (GSE109887). Human brain organoids and primary neuronal models were used to validate the biological relevance of these findings. *AXL* and *ITGB1* were finally selected as key genes for RTK pathway in AD and were significantly increased in AD.

## Key Gene Signatures Reveal Alzheimer’s Disease Diagnostic Potential

Alzheimer disease (AD) is the most common type of dementia, characterized by progressive neurodegeneration. Because early brain changes can precede symptoms, the researchers focused on transcriptomic markers in the middle temporal gyrus (MTG), a region reported to show early AD-related pathology. They analyzed publicly available transcriptome data from the MTG of people with AD and control individuals. Across two independent MTG cohorts, they found that the most consistent disease-related signals were linked to receptor tyrosine kinase pathways, which mediate cell-to-cell communication. The team then confirmed this receptor tyrosine kinase-focused finding using brain organoids that mimic features of the human brain and primary rat neurons exposed to amyloid-beta. They identified key genes, AXL and ITGB1, as potential markers for AD. The study suggests that ITGB1 and AXL are promising biomarker candidates for early AD.

## Introduction

Alzheimer disease (AD) is the most common type of dementia, characterized by progressive neurodegeneration^1,2^. Pathologically, AD is associated with the accumulation of amyloid-beta (Aβ) plaques and tau protein tangles within the brain^3,4^. However, these features may represent downstream consequences rather than the primary drivers of AD, leaving its precise etiology elusive^1^. Importantly, the early diagnosis of AD is critical for effective intervention, as therapeutic strategies targeting the initial stages of the disease may help slow or prevent neurodegeneration.

The middle temporal gyrus (MTG) is a particularly vulnerable region within the complex neuropathology of AD^5,6^. The MTG is intricately connected to broader neural networks via the anterior-temporal and posterior-medial systems^7^. Notably, AD pathology within the MTG often exhibits accelerated progression compared with other brain regions. Studies have shown that the MTG is among the first regions to exhibit notable pathological changes in early AD, including neuronal loss, Aβ deposition, and synaptic dysfunction. These early alterations underscore the critical role of MTG in detecting AD onset and understanding its progression. Despite its significance, the molecular mechanisms underlying the selective vulnerability of MTG in early AD remain elusive. Recent advancements in spatially resolved transcriptomics have provided novel insights into this complex issue by enabling detailed analysis of gene expression patterns within both AD and control MTG tissues^8^. The study identified distinctive cortical layer marker genes, layer-specific differentially expressed genes (DEGs), and altered co-expression patterns associated with AD pathology^8,9^.

Recent investigations underscore the critical role of cell–cell interactions in AD pathogenesis. This intricate process, involving receptors with extracellular ligand-binding domains and intracellular signaling domains, is fundamental to normal brain development and function. Perturbations within this intricate signaling network are increasingly recognized as contributing to the neuropathological hallmarks of the disease^10^. Receptor tyrosine kinases (RTKs) are a pivotal class of transmembrane receptors that orchestrate cell-to-cell communication^11^. These integral membrane proteins possess extracellular ligand-binding domains and intracellular tyrosine kinase catalytic domains^12^. RTKs activate a diverse array of signaling pathways governing essential cellular processes, including survival, differentiation, growth, and migration. Specifically, some studies have suggested the potential role of the RTK pathway in amyloid processing^13^, suggesting a potential role in AD pathogenesis. Furthermore, RTK signaling is intricately connected to other critical pathways, including PI3K–AKT and MAPK cascades, both of which have been implicated in neurodegeneration. Our subsequent analyses therefore explore potential interactions between RTKs and these downstream signaling pathways in AD. Consequently, elucidating the precise mechanisms by which RTK dysregulation contributes to AD progression is imperative for advancing therapeutic strategies.

This study identifies important DEGs of the RTK pathway in the brains of patients with AD by conducting an exploratory transcriptome analysis in two independent public cohorts. Through logistic regression analysis followed by receiver operating characteristic (ROC) curve analysis, we confirmed the diagnostic power of disease-associated genes (DAGs) in AD. Finally, we validated our results by using induced pluripotent stem cell (iPSC)-derived brain organoids and primary rat neurons. Our results highlight RTK pathways and their genetic signatures in AD, offering novel insights and identifying *AXL* and *ITGB1* as potential biomarkers and promising pathologically relevant regulatory factors for AD.

## Methods

### GEO database and cohorts

We leveraged data from two distinct cohorts retrieved from the Gene Expression Omnibus (GEO) database: GSE84422 (ref. ^14^) and GSE109887 (ref. ^15^). Cohort 1 (GSE84422) evaluated molecular signatures and network differences related to brain regional vulnerability in AD. Cohort 2 (GSE109887) examined epigenetic differences between patients with AD and age-matched controls in the MTG. To compare the transcriptome profiles across these cohorts, we utilized the GEO2R analyzer (https://www.ncbi.nlm.nih.gov/geo/geo2r), a widely recognized tool for identifying DEGs in GEO data sets (https://www.ncbi.nlm.nih.gov/geo/). Our objective was to pinpoint genes exhibiting significant expression disparities between the cohorts. We conducted GEO2R analysis for each cohort independently, scrutinizing gene expression profiles across different experimental groups. Rigorous filtering criteria, including adjusted *P*-values and log fold change (FC) values, were applied to ascertain statistically significant DEGs. Following DEG identification in both cohorts, we identified genes displaying differential expressions in both data sets. These overlapping DEGs were subjected to further analysis to unveil consistent expression patterns across cohorts. DEGs were stratified into upregulated and downregulated categories based on their expression profiles within each cohort, providing valuable insights into directional expression changes across the cohorts. From our comparative assessment of the two cohorts, we identified a subset of DEGs representing genes consistently differentially expressed across both data sets (oDEGs). These oDEGs constitute pivotal candidates warranting further investigation in our study.

### ToppGene database analysis

We used the ToppGene platform^16^, an advanced tool for gene list enrichment analysis, to validate the DEGs identified in our transcriptome study of the MTG pertinent to AD. By leveraging the GEO2R analyzer and the Reactome gene set (R-HSA-9006934.7), we conducted analyses on two distinct cohorts (Cohort 1: GSE84422, *n* = 58; Cohort 2: GSE109887, *n* = 78). This step ensured the confirmation of the biological relevance and functional importance of the oDEGs (*n* = 18). The validation process benefited significantly from the capabilities of the ToppGene Suite (https://toppgene.cchmc.org/), which offers a comprehensive toolkit for disease-related gene selection and gene ontology (GO) analysis, identification of potential pathologically relevant regulatory factors, and gene list refinement. In the course of the ToppGene validation, we processed and analyzed the DEGs from our comparative transcriptome study to perform functional annotation and enrichment analysis. Functional enrichment analysis of GO was performed by ToppGene, and the Kyoto Encyclopedia of Genes and Genomes (KEGG) pathways were run by the DAVID online tool (https://david.ncifcrf.gov/)^17,18^.

### Reactome database analysis

For the validation of the overlapped genes of the RTK pathways (*n* = 18), the selected RTK pathway was sorted by the Reactome database (https://reactome.org/)^19^. Eighteen genes (overlapped oDEGs; ooDEGs) were finally selected from the oDEGs (*n* = 145) in comparison to signaling by RTK pathways in the Reactome database (accession no. R-HSA-9006934.7).

### STRING database analysis

To further explore the interaction among the RTK pathway genes with AD, the STRING database (https://string-db.org/) was used for protein–protein interaction (PPI) network construction. Aβ and tau-related genes (*APP*, *APOE*, *PSEN1*, *PSEN2*, *TREM2*, *MAPT*, *BACE1*, and *BACE2*) from genome-wide association studies were added to the 18 ooDEGs, and the network was predicted. Six AD DAGs were finally selected and used for logistic regression analysis and ROC curve analysis.

### Generation of iPSC-derived brain organoids

The human iPSC-derived cortical organoids were generated according to our previous reports^20–22^. A commercialized iPSC line (BIONi010-C) from the European Bank for iPSCs was used for this study. To generate embryoid bodies (EBs), iPSCs were detached by ReLeSR (ST05872, StemCell Technologies) and then loaded into AggreWell plate 800 (34811, StemCell Technologies) with AggreWell EB Formation Medium (ST05893, Stemcell Technologies) containing the ROCK inhibitor, Y-27632. EBs were maintained in AggreWell EB Formation Medium (ST05893, StemCell Technologies) on an AggreWell plate 800 (34811, StemCell Technologies). The next day, the medium was replaced with AggreWell EB Formation Medium (ST05893, StemCell Technologies). From day 2 to day 5, EBs were maintained with the medium composed of DMEM/F-12 containing GlutaMAX (10565018, Gibco), 20% KnockOut Serum Replacement (A3181501, Gibco), 1% MEM Non-Essential Amino Acids Solution (11140050, Gibco), 0.1 mM 2-mercaptoethanol (21985023, Gibco), 100 U/ml penicillin and 100 µg/ml streptomycin (P4333, Merck, Darmstadt, Germany), dorsomorphin (10 µM; P5499, Merck), and SB-431542 (10 µM; 1614, TOCRIS). On day 6, EBs were transferred to a petri dish with a medium composed of Neurobasal-A Medium (10888-022, Gibco), B-27 Supplement minus vitamin A (12587010, Gibco), 100 U/ml penicillin and 100 µg/ml streptomycin, GlutaMAX (10565018, Gibco) Matrigel basement membrane matrix (354234, Corning), 20 ng/ml epidermal growth factor (EGF; AF-100-15, PeproTech), and 20 ng/ml fibroblast growth factor basic (bFGF; 100-18B, PeproTech). From day 7 to day 24, EBs were seeded in 96-well clear round bottom ultra-low-attachment plates (7007, Corning) and maintained with the medium, which was changed every 2 days. From day 25 to day 42, organoids were cultured in Neurobasal-A Medium (10888-022, Gibco), B-27 Supplement minus vitamin A (12587010, Gibco), 100 U/ml penicillin and 100 µg/ml streptomycin, GlutaMAX (35050-061, Gibco), 0.5% (v/v) Matrigel basement membrane matrix (354234, Corning), brain-derived neurotrophic factor (450-02, PeproTech), and 20 ng/ml neurotrophin-3 (450-03, PeproTech), with the medium changed every 2 days. From day 43, the matured organoids were maintained in the medium without growth factors. On day 62, the organoids were used for performing reverse transcription–quantitative PCR (RT–qPCR) and western blotting.

### Aβ preparation

We used synthetic Aβ dissolved in dimethyl sulfoxide and Dulbecco’s phosphate-buffered saline (DPBS) at 100 µM as a stock solution for monomeric Aβ (mAβ) preparations. For brain organoid experiments, we diluted the stock solution in an organoid culture medium or DPBS to achieve a final mAβ concentration of 2 µM. Oligomeric Aβ (oAβ) was generated by incubating the mAβ at 4 °C for 18–24 h. Fibrillar Aβ was generated by incubating the mAβ at 37 °C for 24 h. After incubation, each preparation was diluted to 2 µM in DPBS immediately before use.

### Reverse transcription–quantitative PCR (RT–qPCR)

Total RNAs were isolated from day 62 organoids using RNeasy Mini Kit (74104, Qiagen). RNA concentrations and purity were measured with a NanoDrop spectrophotometer. About 18 ng of RNA was reverse-transcribed using the High-Capacity RNA-to-cDNA kit (4387406, Applied Biosystems) to produce cDNA. RT–qPCR was performed using SYBR^®^ Green Master Mix (A66732, Thermo Fisher Scientific) according to the manufactured protocol on a Quanti-studio™ 3 System (A28567, Applied Biosystems). Gene expression levels of *AXL*, *CAV1*, *GAPDH*, *GFAP*, *ITGB1*, *NRG1*, and *RHOA* were measured using the primer sequences (Supplementary Table 1). ΔΔ Ct method was applied to normalize expression levels of each gene to that of *GAPDH*.

### Western blotting

The western blot experiment was performed to confirm the increased expression of AXL, p-AKT, p-ERK, and p-MARK in AD model organoids^23^. The organoids were treated with 2 µM of Aβ for 1 day. Briefly, the organoids were lysed with RIPA lysis buffer containing a protease/phosphatase inhibitor cocktail for 1 h on ice. The membrane-enriched western blot experiment was performed to verify ITGB1 upregulation at the membrane. The organoids were separated into cytosol and membrane using the MEM-PER™ Plus Membrane Protein Extraction Kit (89842, Thermo Fisher Scientific). Total proteins were quantified using bicinchoninic acid assay, and equal amounts of protein lysates were loaded onto 4–12% Bolt gels (Thermo Fisher Scientific) and electrophoresed for 30 min (200 V, 3000 mA). After electrophoresis, the gels were transferred to polyvinylidene difluoride membranes for 60 min (20 V, 3000 mA) and blocked with a blocking solution (skimmed milk, 5%) for 1 h in ice. The primary antibodies used for this experiment included the human AXL antibody (AF154, Bio-techne, diluted 1:1500 in Tris-buffered saline with 0.1% Tween 20 (TBST)), anti-integrin beta 1 antibody (ab179471, Abcam, diluted 1:1500 in TBST), anti-GAPDH antibody (ab9485, Abcam, diluted 1:1000 in TBST) as a loading control, phospho-Akt (Thr308) antibody (9275S, Cell Signaling Technology, diluted 1:1500 in TBST), Akt antibody (9272S, Cell Signaling Technology, diluted 1:1500 in TBST), phospho-p38 MAPK (Thr180/Tyr182) (D3F9) XP® (4511S, Cell Signaling Technology, diluted 1:1500 in TBST), p38 MAPK antibody (9212S, Cell Signaling Technology, diluted 1:1500 in TBST), phospho-p44/42 MAPK (Erk1/2) (Thr202/Tyr204) (9101S, Cell Signaling Technology, diluted 1:1500 in TBST), p44/42 MAPK (Erk1/2) antibody (4695S, Cell Signaling Technology, diluted 1:1500 in TBST) and the β-amyloid (D54D2) XP (8243T, Cell Signaling Technology, diluted 1:1500 in TBST). For the membrane-enriched western blot experiment, the primary antibodies used were anti-integrin beta 1 antibody (ab179471, Abcam, diluted 1:1500 in TBST), GAPDH antibody (ab9485, Abcam, diluted 1:1000 in TBST) as a loading control, β-amyloid (D54D2) XP (8243T, Cell Signaling Technology, diluted 1:1500 in TBST), and Anti-Sodium Potassium ATPase antibody (ab76020, Abcam, diluted 1:1500 in TBST) as a plasma membrane loading control. The membranes were incubated with these primary antibodies for 18 h at 4 °C. Following this, the membranes were incubated with secondary antibodies for 1 h at room temperature (RT). The secondary antibodies used were rabbit anti-goat IgG H&L-horse radish peroxidase (HRP) (ab6741. Abcam) and goat anti-rabbit IgG H&L-HRP (ab6721, Abcam), which were diluted in blocking solution (3.5% skimmed milk) at a ratio of 1:2500. A bio-imaging analyzer (iBright imaging system, Thermo Fisher Scientific) was used to visualize protein bands, and MULTI-GAUGE software (Fujifilm Corporation) was used for quantification.

### Quantification of secreted proteins in the conditioned medium

iPSCs were generated from five normal controls and five patients with AD and used for the generation of brain organoids (IRB no. SKKU IRB 2024-10-020). The levels of beta-amyloids (1–42, cat: 27711, IBL; 1–40, cat: 27713, IBL), phosphorylated tau (pT181, cat: KHO0631, Invitrogen), and total tau (KHB0041, Invitrogen) in the conditioned medium of brain organoids (day 100) were quantified using enzyme-linked immunosorbent assay, following manufacturer’s instructions^20^. Briefly, to assess protein secretion levels under serum-deprived conditions, the culture medium was replaced with Opti-MEM, and samples were incubated at 37 °C for 12 h. The collected medium was centrifuged at 3000×*g* for 10 min at 4 °C and the protein levels were measured. The same samples were also subjected to bicinchoninic acid analysis, and the results were normalized to total protein content.

### RNA sequencing analysis

To validate the trends in DAGs and to identify relevant pathways, we performed RNA sequencing analysis using data from our previous study (NCBI accession no. PRJNA678865; https://www.ncbi.nlm.nih.gov/bioproject/678865), following the same methodology^20^. We also performed GO analysis with the ToppGene database.

### Primary neuron culture

Primary rat cortical neurons derived from embryonic day 18 Sprague Dawley fetal rats of either sex were prepared as described previously^24^. Briefly, the prefrontal cortex was dissected, dissociated with papain (Worthington Biochemical Corporation), and resuspended in minimal Eagle’s medium (MEM, Invitrogen) supplemented with 0.6% glucose, 1 mM pyruvate, 2 mM L-glutamine, and 10% fetal bovine serum (Hyclone) and plated on poly-D-lysine-coated glass coverslips in 60 mm Petri dishes. Four hours after plating, the medium was replaced with a neurobasal medium (Invitrogen) supplemented with 2% B-27 (Invitrogen) and 0.5 mM L-glutamine. Neurons were fixed on days in vitro 19–21.

### Immunocytochemistry

Primary neurons were fixed with 4% (w/v) paraformaldehyde and 4% (w/v) sucrose dissolved in PBS, pH 7.4 for 15 min at RT, and subsequently permeabilized with 0.25% Triton X-100 in PBS for 3 min at RT. The cells were then blocked for 1 h at RT in 10% (w/v) bovine serum albumin. Cells were incubated at 4 °C overnight with primary antibodies, then washed in PBS, and incubated with secondary antibodies for 1 h at RT. Primary antibodies used for immunocytochemistry were anti-integrin beta 1 (ab179471, Abcam), anti-Axl (AF154, Bio-techne), anti-MAP2 (118 002 and 118 006, Synaptic Systems), and anti-PSD95 (MA1-045, Invitrogen). Alexa Fluor™-647/568/488/405-labeled secondary antibodies were purchased from Thermo Fisher Scientific.

### Confocal imaging and analysis

Images were acquired using a spinning disc confocal microscope (ECLIPSE Ti-E, Nikon) equipped with an oil immersion objective lens (Plan Apo 40× NA 1.30) and a Neo sCMOS camera (Andor Technology) at RT. A frame size 1196 × 1196 was used for all images, resulting in an XY pixel size of 162.5 nm. The *z*-step size was 300 nm, with 13–15 steps per *Z*-stack. A 95% laser power was used for the 405/488/561/647 lasers. Imaris software (Bitplane AG) was used for measuring AXL and ITGB1 intensity. The surface function was used to generate volumes representing MAP2 and PSD95. The AXL or ITGB1 intensity masked with MAP2 or PSD95 was measured.

## Results

### Overall study procedures and demographic characteristics of participants

**Fig. 1:**
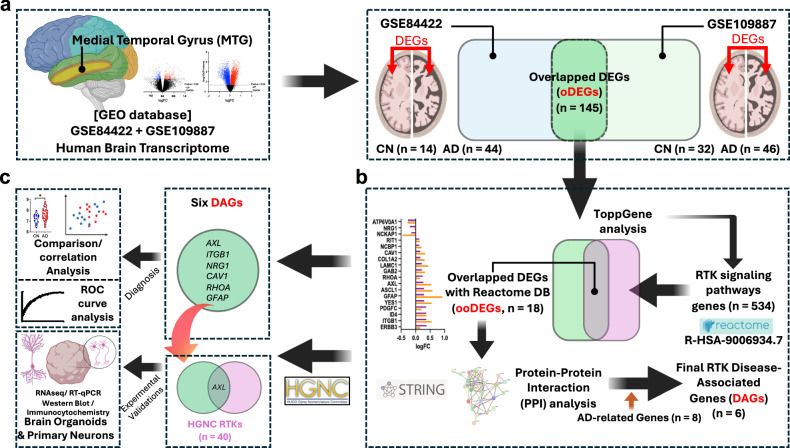
Overall study procedures and demographic characteristics of participants. **a** Comparing transcriptomic data of the middle temporal gyrus (MTG) in human post-mortem brain samples using two independent public data sets from the GEO database (cohort 1, GSE84422, *n* = 58; cohort 2, GSE109887, *n* = 78) using GEO2R analyzer. **b** Finding the overlapped DEGs (*n* = 18) using ToppGene and validation with the Reactome gene set (*n* = 534) (R-HSA-9006934.7). The significant disease-associated genes (DAGs, *n* = 6) directly linked with the main AD genes, were finalized using the STRING database. **c** ROC curve analysis (six DAGs) and experimental validations with iPSC-derived brain organoids and primary rat neurons. AD Alzheimer disease, CN cognitively normal, DAG disease-associated gene, DEG differentially expressed gene, FC fold change, HGNC HUGO Gene Nomenclature Committee, MTG middle temporal gyrus, PPI protein–protein analysis, ROC receiver operating characteristic, RTK receptor tyrosine kinase, RT–qPCR reverse transcription–quantitative PCR.

The flow chart of the experimental procedures is presented in Fig. 1. Two independent cohorts (cohort 1, USA cohort for brain transcriptome analysis (*n* = 58); cohort 2, European cohort for brain transcriptome analysis (*n* = 78)) were included in this study. Cohort 1 included 14 cognitively normal (CN) individuals and 44 with individuals with AD. Cohort 2 included 32 CN individuals and 46 with AD (Table 1). After screening with the thresholds of |log 2FC| > 0 and *P*-value < 0.05, 145 oDEGs were identified (Fig. 1a). The oDEGs were analyzed with ToppGene and the most significant RTK pathway candidates were selected. The oDEGs were examined again in the Reactome database (R-HSA-9006934.7) for RTK signaling pathways, and 18 ooDEGs were identified (Fig. 1b). Then, PPI network analysis was performed using the STRING database combined with amyloid/tau-related genes from genome-wide association studies and six genes were selected. ROC curve analysis comprising these six genes showed a significant association of RTK pathways with AD (Fig. 1c). For biological validation, western blot analysis and RNA-sequencing analysis were performed by using iPSC-derived brain organoids, and immunocytochemistry was performed by using primary rat neurons (Fig. 1c).

**Table 1:** Demographic and clinical characteristics of participants in cohort 1 (GSE84422) and cohort 2 (GSE109887).

GSE84422	CN (14)	AD (44)	Total (58)	*P*-value
**Sex,** ***n*** **(%)**				
Female	8 (13.8%)	30 (51.7%)	38 (65.5%)	0.4491^a^
Male	6 (10.3%)	14 (24.1%)	20 (34.5%)	
**Age (years)**	80.93 ± 3.48	87.36 ± 1.14		0.0255^b^
**Race**				
White	10	34	44	0.2473^a^
Black	2	9	11	
Hispanic	1	1	2	
Asian	1	0	1	
**Braak neurofibrillary tangle score**				<0.0001^a^
0	1	0		
I/II	10	5		
III/IV	2	22		
V/VI	1	17		
**Clinical dementia rating**				<0.0001^a^
0	10	2		
0.5	4	6		
1	0	7		
2	0	7		
≥3	0	22		
**Average neuritic plaque density**				<0.0001^*^
0	10	1		
>0	4	43		
**Sum of CERAD rating scores in multiple brain regions**				<0.0001^a^
0	10	1		
>0	4	43		
**Sum of neurofibrillary tangle density in multiple brain regions**				0.0016^a^
0	3	0		
>0	11	44		

GSE109887	CN (32)	AD (46)	Total (78)	*P*-value
**Sex,** ***n*** **(%)**				
Female	16 (20.5%)	24 (30.8%)	40 (51.3%)	0.8501^a^
Male	16 (20.5%)	22 (28.2%)	38 (48.7%)	
**Age (years)**	84.6 ± 0.96	85.1 ± 0.91		0.729^b^

*AD* Alzheimer disease, *CN* cognitively normal. ^a^*P*-values from χ^2^ test.

^b^*P*-values from *t*-test.

### Human brain transcriptomic analysis revealed RTK pathways upregulated in patients with AD

**Fig. 2:**
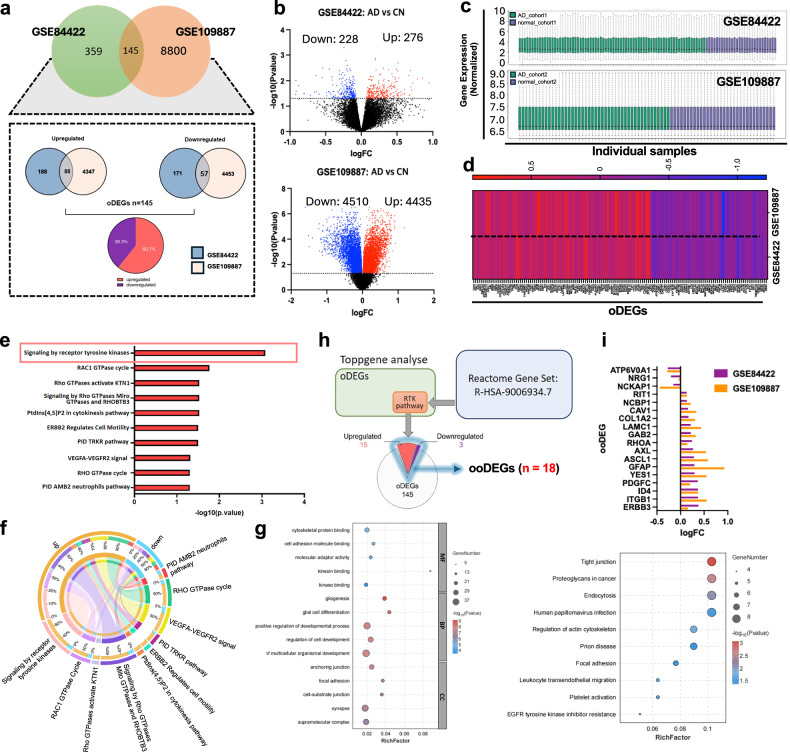
Comparative human transcriptomic analysis in the middle temporal gyrus of Alzheimer disease brains. **a** Venn diagram shows the oDEGs between GEO84422 and GEO109887 data sets and percentages of upregulated and downregulated genes. **b** Volcano plots of DEGs in GEO84422 and GEO109887, respectively. Red represents the upregulated genes, and blue represents the downregulated genes. The thresholds of raw *P*-value < 0.05 for cohort 1 and adjusted *P*-value < 0.05 for cohort 2 were used. **c** Box plots of normalized gene expressions across individual samples in the two data sets. **d** Heatmap for displaying gene expression levels of 145 oDEGs in the two data sets. **e** ToppGene (biological process (BP)) analysis shows significant terms related to receptor tyrosine kinases (RTKs). **f** Circos graph showing the correlation between upregulated/downregulated genes and enriched pathways. Circos graph and the bubble diagrams of GO, KEGG were visualized by OmicShare. **g** Enriched KEGG pathways and gene ontology analysis (BP, CC, and MF) of oDEGs. The analytic threshold of enrichment analyses was an FDR-adjusted *P* < 0.05. **h** The number of DEGs overlapping with receptor tyrosine kinases pathway selected with ToppGene and Reactome (R-HSA-9006934.7). **i**, Expression distribution of 18 genes involved in receptor tyrosine kinases pathway. AD Alzheimer disease, CN cognitively normal, DEG differentially expressed gene, RC fold change, oDEG overlapped DEG, ooDEG overlapped DEG overlapped with Reactome gene set, RTK receptor tyrosine kinase.

To investigate the specific molecular factors and pathways related to AD, we used the GSE84422 and GSE109887 data sets from the NCBI GEO database and performed transcriptome analyses of AD human post-mortem brains using the GEO2R analyzer. For cohort 1,504 DEGs (276 upregulated and 228 downregulated) were identified in the GSE84422 data set. For cohort 2, 8,945 DEGs (4,435 upregulated and 4,510 downregulated) were identified in GSE109887 (Fig. 2a,b). We found 145 oDEGs, 88 upregulated and 57 downregulated, between the two cohorts (Fig. 2a). Also, we confirmed that these data sets are normalized (Fig. 2c,d).

To explore the functions and pathways of oDEGs, we carried out functional enrichment analysis of the 145 oDEGs by using ToppFun. ToppGene Suite results showed that the oDEGs from the two data sets were remarkably enriched in RTK pathways with a false discovery rate-corrected *P*-value of 8.3110 × 10^−4^, followed by the RAC1 GTPase cycle, and Rho GTPases activate KTN1 (Fig. 2e). The top 10 enriched pathways and related upregulated/downregulated genes are shown in Fig. 2f. The 145 oDEGs were also imported into the DAVID online tool (https://david.ncifcrf.gov/) for KEGG pathway analysis (Fig. 2g). The top 5 significant KEGG pathways of the DEGs were tight junction, proteoglycans in cancer, endocytosis, regulation of the action cytoskeleton, and leukocyte transendothelial migration. We examined the RTK pathway-related genes from ToppFun analysis, and 18 genes were extracted. Subsequently, we validated these genes by analyzing the signaling by RTK gene set in Reactome (accession no. R-HSA-9006934.7). We finally obtained 15 upregulated oDEGs and 3 downregulated oDEGs, which overlapped with the RTK pathway in the Reactome gene set and defined them as ooDEGs (Fig. 2h,i). To determine whether RTK dysregulation extends beyond the MTG, we analyzed a publicly available hippocampal transcriptome data set (GSE1297 and GSE48350). Our analysis identified significance of RTK pathway enrichment among the oDEGs in the hippocampus. On the basis of these results, we hypothesize that the RTK dysregulation may also be relevant in other AD-vulnerable brain regions (Supplementary Fig. 1).

### Molecular network analysis shows essential genetic regulation of RTK pathways in patients with AD

**Fig. 3:**
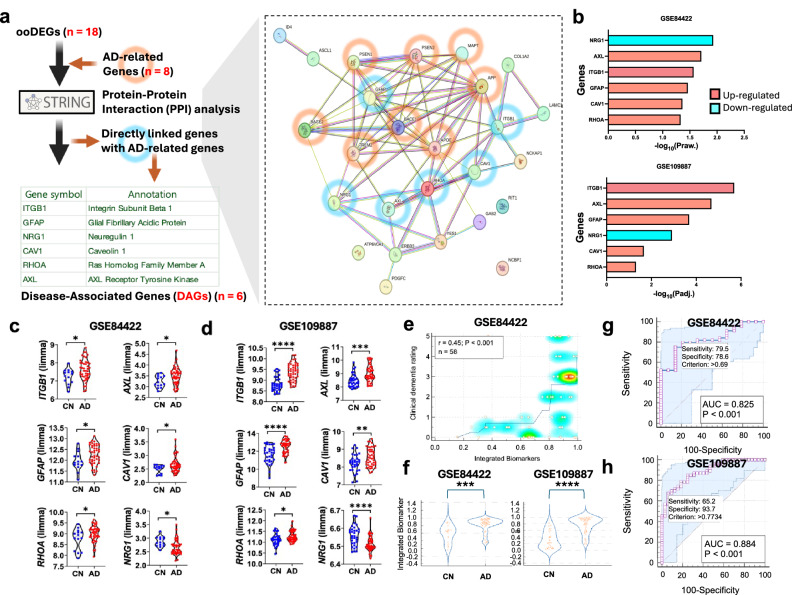
Molecular network shows essential genes for receptor tyrosine kinase pathways in Alzheimer disease. **a** Protein–protein interaction (PPI) analysis to finalize disease-associated genes (DAGs). Cyan circles represent six DAGs, and orange circles represent eight AD-related genes. **b** Upregulation and downregulation tendency of DAGs. **c**,**d** Comparison of gene expression (normalized by limma in GEO2R) between CN and AD for both cohorts. Unpaired *t*-test, *P < 0.05, **P < 0.01, ***P < 0.001, and ****P < 0.0001. **e** Scatter diagram (isotonic regression curve) between the integrated biomarker values (logistic regression analysis with six DAGs) and clinical dementia rating scores. ***P < 0.001 and *r* (coefficient) by Pearson’s correlation analysis. **f** Comparison of integrated biomarker values between CN and AD. Unpaired *t*-test, ***P < 0.001 and ****P < 0.0001. **g**,**h** ROC curve analysis following the logistic regression analysis for six DAGs. AD Alzheimer disease, AUC area under the curve, CN cognitively normal, DAG disease-associated gene, ooDEG overlapped DEG overlapped with Reactome gene set, Path pathway database, ROC receiver operating characteristic.

We found that the RTK pathways are upregulated in AD, but it remains unclear how these genes are interconnected and how they are related to AD-associated genes. Using the STRING database, an analytic tool for functional protein association networks, we established a molecular network of RTK pathway gene sets (Fig. 3a) combined with amyloid/tau-related genes (*APP*, *APOE*, *PSEN1*, *PSEN2*, *TREM2*, *MAPT*, *BACE1*, and *BACE2*). Through STRING database-based PPI analysis, six candidate hub genes were identified from the network: *ITGB1*, *AXL*, *GFAP*, *NRG1*, *CAV1*, and *RHOA*. Interestingly, *ITGB1*, *AXL*, *GFAP*, *CAV1*, and *RHOA* were directly associated with the *APOE* gene, and *ITGB1*, *GFAP*, *CAV1*, and *RHOA* were directly associated with the *APP* gene. Moreover, *GFAP* had the most interaction genes including *APOE*, *APP*, *MAPT*, *PSEN1*, *PSEN2*, and *TREM2*. *AXL* was also directly associated with the *TREM2* gene and *NRG1* was directly associated with the *BACE1* and *BACE2* genes. Among the six genes, *NRG1* was downregulated in AD and the others were upregulated in AD (Fig. 3b). We confirmed that the levels of the DAGs (normalized counts by limma in GEO2R) were significantly different between CN and AD (Fig. 3c,d). Subsequently, we integrated six DAGs by logistic regression analysis and performed Pearson’s correlation analysis with isotonic regression curve (Fig. 3e). We found a significant correlation between the integrated biomarker values and clinical dementia rating scores and confirmed the significantly different levels of the integrated biomarker values between CN and AD (Fig. 3e,f). To further examine the full spectrum of AD progression, we re-analyzed the GSE84422 data set specifically by AD stage. Our results revealed no RTK pathway activation in possible AD, whereas both probable and definite AD stages showed clear RTK activation (Supplementary Fig. 2).

### ROC curve analysis with DAGs for the discrimination between CN and AD

As the levels of DAGs were significantly different between CN and AD, ROC curve analysis followed by logistic regression analysis was performed to calculate the diagnostic power for AD. For both GSE84422 and GSE109887 cohorts, the area under the curve values were more than 0.8 and *P*-values were under 0.001 (Fig. 3g,h); this indicates that our combinative biomarker with six DAGs has strong discrimination power for AD diagnosis.

### Validation of DAGs by using brain organoids from patients with AD

**Fig. 4:**
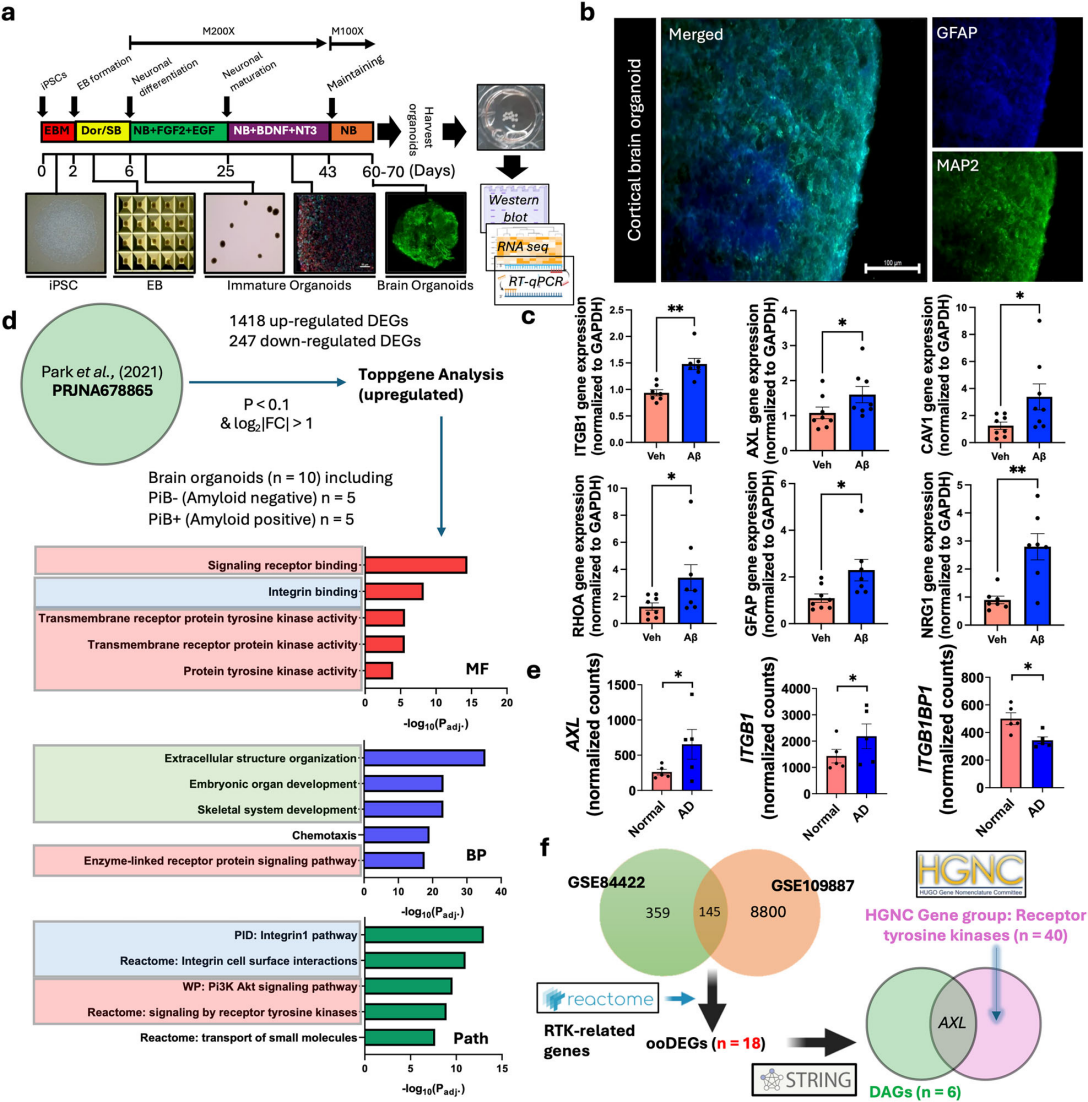
Identification of the final key DAG using iPSC-derived cerebral organoid-derived RT–qPCR and RNA sequencing. **a** Generation of iPSC-derived cerebral organoids. **b** Immunohistochemistry for validation of iPSC-derived cerebral organoids. Anti-GFP (astrocyte) and anti-MAP2 (neuron) antibodies were used. Scale bar, 100 μm. **c** RT–qPCR analysis of DAG expression levels in vehicle-treated (Veh) and Aβ-treated organoids. Each, *n* = 8; *P < 0.05 and **P < 0.01; Mann–Whitney test. Outliers were assessed using GraphPad’s outlier calculator (QuickCalcs Grubbs test; *P = 0.05 and excluded. **d** RNA-sequencing and gene ontology (GO) analysis using the PRJNA678865 GEO data set. Ten brain organoids were used for the data set, and the criteria for differentially expressed genes (DEGs) were *P* < 0.1 and log_2_FC > 1 or <−1. The upregulated 1418 DEGs were used for GO analysis. Red boxes indicate pathways related to receptor tyrosine kinase pathways (receptor binding, kinase activity, PI3K–AKT pathways, and so on), green box indicates pathways related to synaptic developments or structures, and sky-blue boxes indicate pathways related to integrin (integrin binding, integrin 1 pathway, integrin interaction, and so on). **e** Comparison of the levels of genes between normal organoids and AD organoids. Among DAGs, *AXL* and *ITGB1* were the significant DEGs in our RNA-sequencing data set (*P < 0.1 by limma). **f** The overlapping result between six DAGs and the HGNC gene group (RTK). *AXL* was the only RTK itself among DAGs. Aβ amyloid-beta, AD Alzheimer disease, BP biological process, DAG disease-associated gene, DEG differentially expressed gene, EB embryoid body, FC fold change, HGNC HUGO Gene Nomenclature Committee, iPSC induced pluripotent stem cell, MF molecular function, ooDEG overlapped DEG overlapped with Reactome gene set, Path pathway database, PiB Pittsburg compound B, RNA seq RNA sequencing, RTK receptor tyrosine kinase, RT–qPCR reverse transcription–quantitative PCR, Veh vehicle treated.

**Fig. 5:**
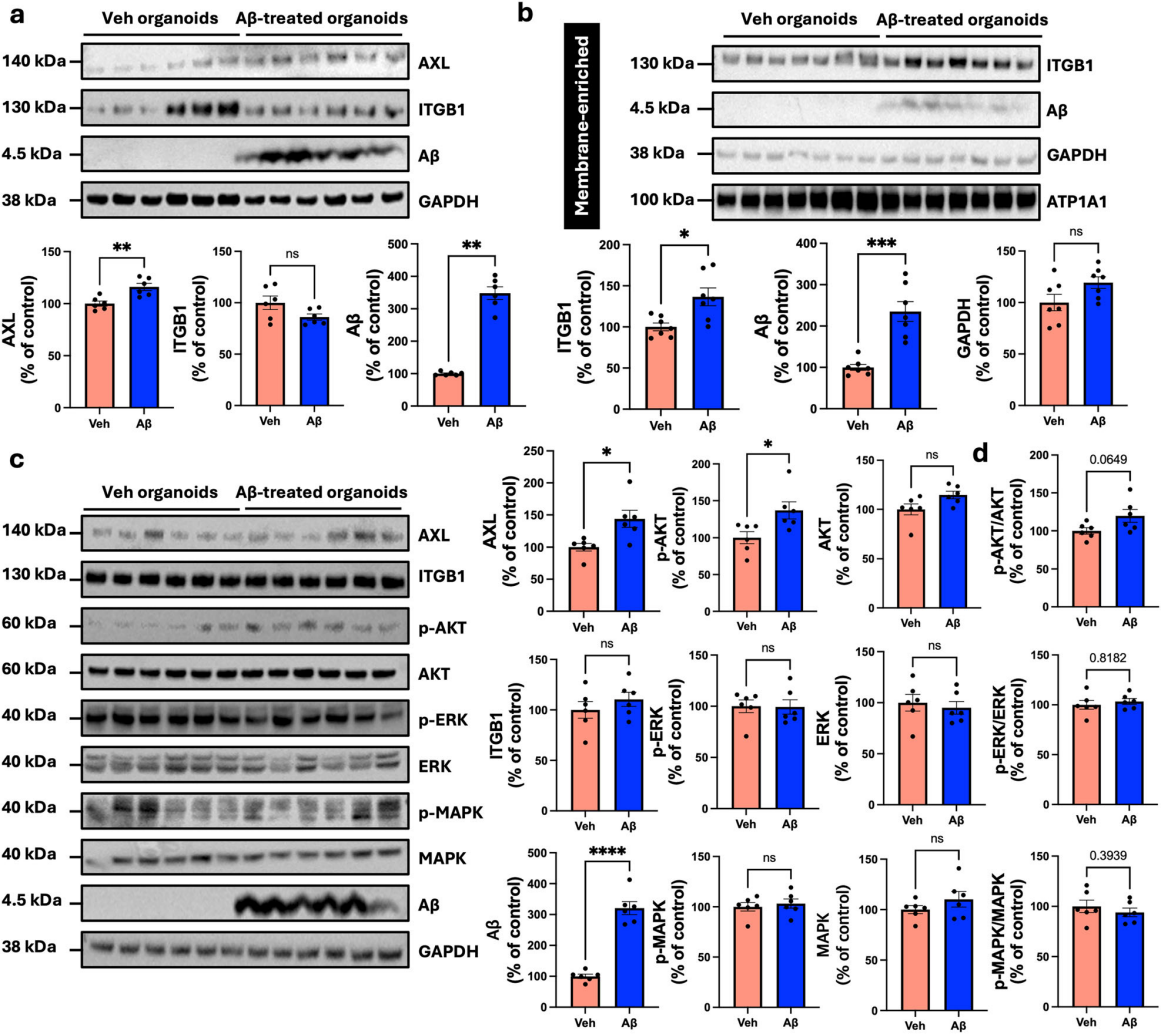
Western blot analysis of AXL, ITGB1, MAPK/ERK, and PI3K–Akt pathway levels in vehicle-treated and Aβ-treated organoids. **a** Western blot analysis for AXL and ITGB1 between vehicle-treated and Aβ-treated organoids. Each, *n* = 6; **P < 0.01 by Mann–Whitney test. **b** Membrane-enriched western blot analysis of ITGB1 between vehicle-treated and Aβ-treated organoids. Each, *n* = 7; *P < 0.05 and ***P < 0.001 by Mann–Whitney test. **c** Western blot analysis for phosphorylated MAPK (p-MAPK) and phosphorylated ERK (p-ERK) and phosphorylated Akt (p-Akt) between vehicle-treated and Aβ-treated organoids. *P < 0.05 and ****P < 0.0001 by Mann–Whitney test. **d** Quantification of p-MAPK and p-ERK and p-Akt levels, normalized to total MAPK (t-MAPK), total ERK (t-ERK), and total Akt (t-Akt) expression. *P*-values were determined by the Mann–Whitney test. Aβ amyloid-beta, ns no significance, Veh vehicle-treated.

For experimental validation of the DAGs, iPSC-derived brain organoids were created. We adopted a published protocol with minor modification^25^ (Fig. 4a). We confirmed the expression of a mature neuron marker (MAP2) and an astrocyte marker (GFAP) in the brain organoids (Fig. 4b). To validate changes in the DAGs, we performed RT–qPCR to quantify gene expression in brain organoids treated with mAβ. The aggregation state of our Aβ preparations was verified by western blot analysis, which showed the expected size distributions (Supplementary Fig. 3). Consistent with our DAG-based transcriptomic analyzing, the expression of *AXL*, *ITGB1*, *CAV1*, *GFAP*, and *RHOA* increased upon Aβ exposure. *NRG1* also showed an increase rather than a decrease (Fig. 4c). Enzyme-linked immunosorbent assay revealed a significant increase of Aβ/tau protein in the conditioned media of AD-derived brain organoids (Supplementary Fig. 4). Interestingly, RNA-sequencing analysis with our brain organoids (five CN and five AD; the same cohort used by Park et al.^20^) showed significantly enriched RTK-related pathways in GO terminologies (highlighted in red), integrin-related pathways (highlighted in blue), and organ development and organization (highlighted in green) (Fig. 4d). Interestingly, the PI3K–AKT signaling pathway was highly associated with AD brain organoids, which is highly related to AXL and RTK pathways. Also, the levels of *AXL*, *ITGB1*, and its binding protein *ITGB1BP1* (two genes out of the six DAGs) were also significantly different between CN and AD in our RNA-sequencing data from brain organoids (Fig. 4e and Supplementary Fig. 5). We found that the *AXL* gene was the only member of the RTK family identified by the HGNC gene group (Fig. 4f). To probe early AD-like changes in AXL and ITGB1, we treated brain organoids with mAβ. We performed western blot analysis and validated that Aβ induces significantly increased levels of AXL in the brain organoid lysates (Fig. 5a). This finding supports the relevance of Aβ-related toxicity to amyloid plaque pathology, emphasizing its mechanistic link to RTK signaling in AD. By contrast, a parallel western blot for ITGB1 in the same organoid lysates showed no significant change (Fig. 5a). As ITGB1 is a plasma membrane protein, we performed membrane-enriched western blot analysis. In the membrane-enriched samples, mAβ treatment significantly increased ITGB1 levels in brain organoids membrane-enriched samples (Fig. 5b). To determine whether Aβ selectively modulates AXL-dependent RTK signaling, we conducted western blot analysis using brain organoid lysates. The result revealed a significant increase in AXL and phosphorylated-AKT levels following 2 µM mAβ treatment, whereas p-MAPK and p-ERK levels remained unchanged (Fig. 5c). To further characterize downstream RTK signaling, we performed a STRING-based PPI analysis focusing on AXL and ITGB1. Using KEGG pathway annotation, we retrieved gene sets involved in MAPK/ERK (hsa04010), JAK/STAT (hsa04630), and PLCγ/PKC (hsa04070) pathways as well as PI3K–AKT (hsa04151). We then assessed high-confidence (score ≥ 0.7) interactions of AXL and ITGB1 within these pathways (Supplementary Fig. 6a). Comparative analysis with oDEGs revealed that AXL and ITGB1 formed four direct PPI links in the PI3K–AKT pathway, two in the MAPK/ERK pathway, and one in the JAK/STAT pathway, whereas the PLCγ/PKC pathway showed only indirect interactions with AXL or ITGB1. These results support a selective enrichment of AXL and ITGB1 interactions in the PI3K–AKT axis under AD-like conditions (Supplementary Fig. 6b,c). These findings suggest that Aβ triggers a predominant activation of the AXL–PI3K–AKT axis rather than a general stress response (Fig. 5d), thereby implicating a mechanistic link between Aβ toxicity and RTK signaling under AD-like conditions.

### Validation of Axl RTK expression levels in AD by using primary cortical neurons

**Fig. 6:**
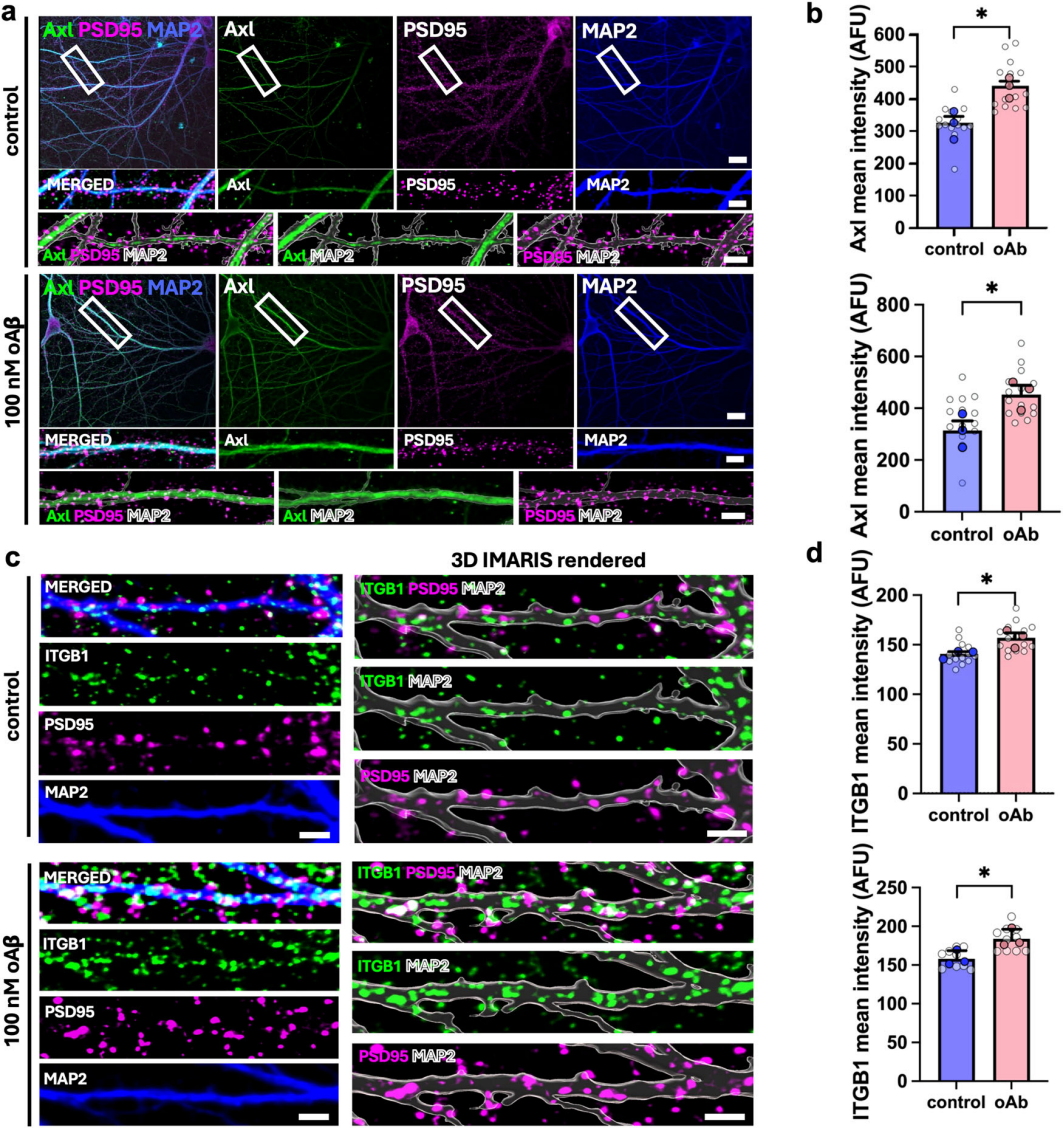
Experimental validation of oAβ-induced Axl and ITGB1 upregulation in primary rat cortical neurons. **a** Representative immunocytochemistry images of primary cortical neurons labeled for Axl (green), PSD95 (magenta), and MAP2 (blue) after 24 h incubation with or without 100 nM oAβ. The lower panels in each group show enlarged dendrites with 3D volume-rendered images using Imaris. Scale bars, 2 μm and 5 μm. **b** Quantification of Axl intensity in MAP2-positive regions (upper) or PSD95-positive regions (lower). For quantification, regions of interest (ROIs) were selected from four to six individual neurons. Each group included 14–15 ROIs obtained from three independent cultures. Data shown as mean + S.E. Unpaired *t*-test, *P < 0.05. **c** Representative images of primary cortical neurons labeled for ITGB1 (green), PSD95 (magenta), and MAP2 (blue) after 24 h of incubation with or without 100 nM oAβ, followed by immunocytochemistry. The right panels in each group show 3D volume-rendered images using Imaris. Scale bar, 5 μm. **d** Quantification of ITGB1 intensity in MAP2-positive regions (upper) or PSD95-positive regions (lower). For quantification, ROIs were selected on from three to five individual neurons. Each group included 10–15 ROIs obtained from three independent cultures. Data shown as mean + S.E. Unpaired *t*-test, *P < 0.05. oAβ, oligomeric amyloid-beta.

As AXL was the member of the RTK family identified (Fig. 4f), while other DAGs were related to RTK signaling pathways, we further explored the role of AXL in AD. Especially, as our brain organoids used for RNA-sequencing analysis (Fig. 4e,f) were at day 60 in vitro and contained major neuronal populations, we decided to assess Axl levels in AD-conditioned neurons. To investigate the potential association between Axl expression and AD pathology, we assessed neuronal Axl levels following exposure to Aβ oligomers (oAβ), a synaptotoxic species implicated in AD pathogenesis. We performed immunocytochemistry on primary cultured cortical neurons using specific antibodies against Axl, Psd95, and Map2 after the application of 100 nM of soluble oAβ. As expected, we observed a significant increase in the mean intensity of Axl in both Map2-positive (neuronal) and Psd95-positive (synaptic) regions following oAβ treatment (Fig. 6a,b). The increased Axl expression in both neuronal and synaptic regions highlights its potential role in mediating the detrimental effects of oAβ on neuronal function and synaptic integrity. Furthermore, the upregulation of Axl could contribute to the inflammatory processes associated with AD.

### Validation of ITGB1 and postsynaptic function in brain organoids and primary neurons

As we found a notable upregulation of the AXL in organoids (Fig. 4) and elevated expression in primary cortical neurons (Fig. 6a,b) exposed to oAβ, we next investigated the potential involvement of ITGB1. We examined the expression and localization of ITGB1 in both brain organoids and primary cortical neurons. Our immunocytochemical analysis revealed a similar trend to AXL, with a slight increase in ITGB1 levels in both MAP2-positive and PSD95-positive regions (Fig. 6c,d). These results suggest that ITGB1 may have a role in synaptic function, as evidenced by its colocalization with PSD95 at the synapse. The upregulation of ITGB1 in response to oAβ could contribute to the synaptic dysfunction observed in AD.

### Possible signaling pathways related to AXL and ITGB1

Here, we show a graphic summary of a possible mechanism of AXL RTK and ITGB1 (Supplementary Fig. 7) in the progression of AD, especially in the MTG brain region. As RTK modulates integrin alpha/beta 1 subunit via PI3K–AKT signaling pathways and β-site APP cleavage enzyme activity^13^, our results revealed the contribution of AXL RTK, ITGB1, and PI3K–AKT to AD pathogenesis. We speculate that the Axl RTK can accelerate disease severity via a positive feedback loop leading to a vicious reinforcing cycle in AD. We suggest that therapeutic approaches that target RTK pathways should be investigated.

## Discussion

Our study identified six critical DAGs associated with AD in the MTG brain region, an area crucial for language, memory, and sensory integration and notably vulnerable in early AD stages^8,26^. Through comprehensive transcriptome analysis of human post-mortem brain samples and databases including GEO, Reactome, and STRING, we recognized more than 100 oDEGs, with a particular focus on the RTK pathway, which is integral for brain development. The highlighted genes — *NRG1*, *RHOA*, *CAV1*, *AXL*, *GFAP*, and *ITGB1* — have significant roles in neurotransmission, cellular adhesion, and migration, suggesting their pivotal involvement in maintaining or restoring neural function in AD^27,28^. We narrowed down specific targets via RT–qPCR, RNA-sequencing analysis, western blotting, and immunocytochemistry by using both human brain organoids and primary rat cortical neurons, confirming that *AXL* and *ITGB1* are highly associated with AD pathogenesis in the brain.

Although iPSC-derived brain organoids and primary rat neurons do not precisely replicate the MTG, we further analyzed publicly accessible hippocampal transcriptome data sets, another region critically involved in AD pathology. Our findings revealed a similarly enriched RTK pathway signature in the hippocampus, suggesting that RTK dysregulation may extend beyond the MTG to other AD-vulnerable brain areas. Together, these observations emphasize the broader relevance of RTK signaling in AD and validate our use of forebrain organoids and primary neurons to investigate underlying molecular mechanisms.

Importantly, across all three models (MTG tissue, Aβ-treated human cortical organoids, and Aβ-treated primary neurons), we consistently observed upregulation of ITGB1 and AXL, indicating reproducible engagement of RTK pathways under AD-relevant conditions. Prior work has shown that β1-integrin activation in astrocytes and microglia drives FAK phosphorylation, NOX2-dependent ROS production, and inflammatory gliosis in response to Aβ, consistent with an integrin β1–FAK–NOX2 cascade mediating oxidative and inflammatory stress^29^. In our study, the ITGB1 upregulation that we observe in RTK-enriched contexts across AD MTG tissue, Aβ-treated human cortical organoids, and Aβ-treated primary neurons is therefore best interpreted as conceptually aligning with models in which integrin β1 feeds into downstream modules that are also shared with RTK signaling, rather than as direct evidence that RTK activation itself induces ITGB1 expression. Other studies have identified AXL as a plaque-associated RTK in disease-associated microglia, linking it to Aβ plaque recognition and phagocytosis, synaptic pruning, and APOE homeostasis^30–32^, whereas canonical Gas6–AXL models in non-AD systems demonstrate robust PI3K–AKT activation and pro-survival, anti-apoptotic signaling downstream of AXL^33^. Within this framework, our data extend these established mechanisms by showing that ITGB1-centered and AXL-centered RTK pathways are not restricted to glial contexts, but can also be detected across AD MTG tissue, Aβ-treated human cortical organoids, and neuron-containing in vitro systems such as Aβ-treated primary neurons. Consistent with this notion, in our Aβ-treated cortical organoids, upregulation of AXL and ITGB1 was accompanied by a trend toward increased AKT phosphorylation (p-AKT/AKT ratio), whereas MAPK/ERK phosphorylation did not show a comparable change under the same conditions. Together, these observations suggest that, under the specific Aβ paradigms and readouts examined here, PI3K–AKT behaves as a predominant branch within the RTK network, while not excluding the involvement of additional RTK downstream pathways in AD.

Our findings align with existing studies emphasizing the role of RTK pathways in brain function and AD development, underscoring RTK pathways as a promising therapeutic target. However, our research advances the field by isolating specific genes within the MTG that are critically involved in AD pathology, a step further than many studies focusing on broader regions or pathways^6,34,35^. This precise identification of essential genes related to the RTK pathway and their indispensable roles in cerebral development and homeostasis provides a more focused approach to understanding AD mechanisms^10^.

Identifying these six DAGs offers new insights into AD pathology and highlights potential pathologically relevant regulatory factors within the MTG. Understanding the roles of these genes in the RTK pathway and their impact on brain function could lead to novel treatments aimed at restoring pathway competence. The use of public data sets for this analysis demonstrates the potential of shared resources to expedite AD research, offering a cost-effective method for identifying and validating crucial genes involved in disease pathogenesis.

Although our findings offer promising avenues for AD research, they are based on cohort analysis and require further experimental validation to fully confirm roles of these genes in AD pathology. In addition, MTG bulk transcriptome signatures may be influenced by region-specific shifts in astrocyte and microglial proportions in AD. Thus, the observed gene expression changes, including the upregulation of AXL and ITGB1, should be interpreted in light of this potential cell-type confounding. Among our six identified DAGs, we performed experimental validation exclusively on *AXL* and *ITGB1* owing to their overlap with our RNA-sequencing data. However, multiple independent studies have also demonstrated the involvement of NRG1 (ref. ^36^), RHOA^37^, CAV1 (ref. ^38^), and GFAP^39^ in AD pathology. Additionally, although the utilization of publicly accessible data sets offers advantages in terms of resource optimization and broad accessibility, it may impose limitations on the comprehensiveness of data available for analytical purposes. Furthermore, the diagnostic performance of our six DAGs ROC model should be interpreted as preliminary, as no additional experimental validation was performed in the present study. Moreover, the absence of perturbation experiments, for example, knockdown or overexpression of key regulators such as AXL or ITGB1, limits causal interpretation of the observed associations, and such mechanistic studies will be essential in future work. Future studies should validate these findings through experimental models and explore the therapeutic potential of targeting these genes in AD treatment strategies.

In summary, by various bioinformatical analyses and experimental validations, we identified six DAGs and showed a possible mechanism of final targets (AXL RTK pathway and ITGB1) in the progression of AD, especially in the MTG brain region. We speculate that the AXL RTK pathway can accelerate AD pathogenesis via a positive feedback loop with a vicious reinforcing cycle via PI3K–AKT between the AXL RTK pathway and ITGB1. Thus, we believe that therapeutic approaches that target the AXL RTK pathway and ITGB1 should be investigated, to understand disease pathology in the MTG brain region of AD. Although we focused on RTK dysregulation, AD remains a multifactorial disease involving immune, synaptic, and metabolic pathways. Future integrative analyses or multi-omics approaches will be essential to position AXL and ITGB1 within this broader pathogenic network. Nevertheless, we prioritized the RTK pathway because our gene-expression analyses consistently ranked it among the most enriched pathways, underscoring its critical involvement in AD pathogenesis.

## Supplementary information

Supplementary Information
