# Supplementary material for: Human brain and organoid transcriptomes reveal key receptor tyrosine kinase pathways and genetic signatures in Alzheimer's disease
Source: Exp Mol Med. 2026 Apr 15;58(4):1230–41. doi: 10.1038/s12276-026-01684-5 (PMC13144494; doi:10.1038/s12276-026-01684-5)
Supplement: Supplementary file 1 — Supplementary Information [file 12276_2026_1684_MOESM1_ESM.pdf]

Supplementary Information

Human brain and organoid transcriptomes reveal key receptor tyrosine kinase pathways and genetic signatures in Alzheimer's disease

Saewoon Shin, Xiaohui Zhu, Sarnai Amartumur *et al.*,

***Corresponding Authors:**

Sang-Eun Lee, Ph.D., Department of Life Sciences, CHA University, Seongnam, Gyeonggi, 13488, Republic of Korea

Tel: +82-10-9451-0504; E-mail: sangeun@cha.ac.kr

Jong-Chan Park, Ph.D., Department of Biophysics, Sungkyunkwan University, Gyeonggi-do, 16419, Republic of Korea

Tel: +82-31-299-4795; Fax: +82-31-299-4093; E-mail: jongchan@g.skku.edu

Supplementary Fig. 1. Transcriptomic analysis data from the hippocampus

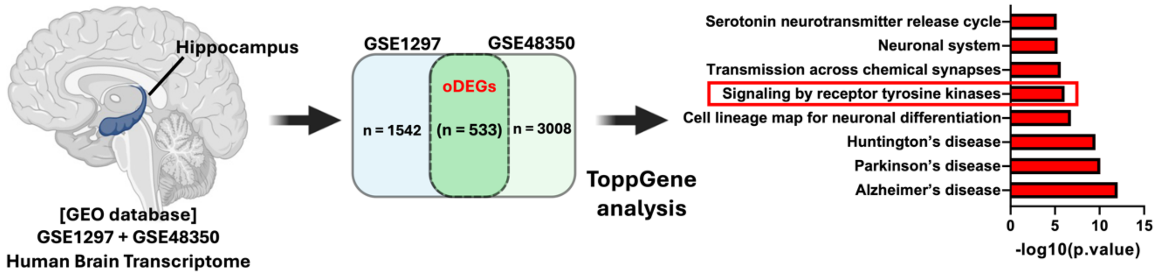

Comparing transcriptomic data of hippocampus in human postmortem brain samples of two independent public datasets in the GEO database (Cohort 3, GSE1297; Cohort 4, GSE48350,) using GEO2R analyzer. Venn diagram shows the oDEGs between GSE1297 and GSE48350 datasets. Toppgene showed significant terms related to receptor tyrosine kinases (RTKs).

Supplementary Fig. 2. Stage-specific activation of the RTK pathway in Alzheimer's disease (AD).

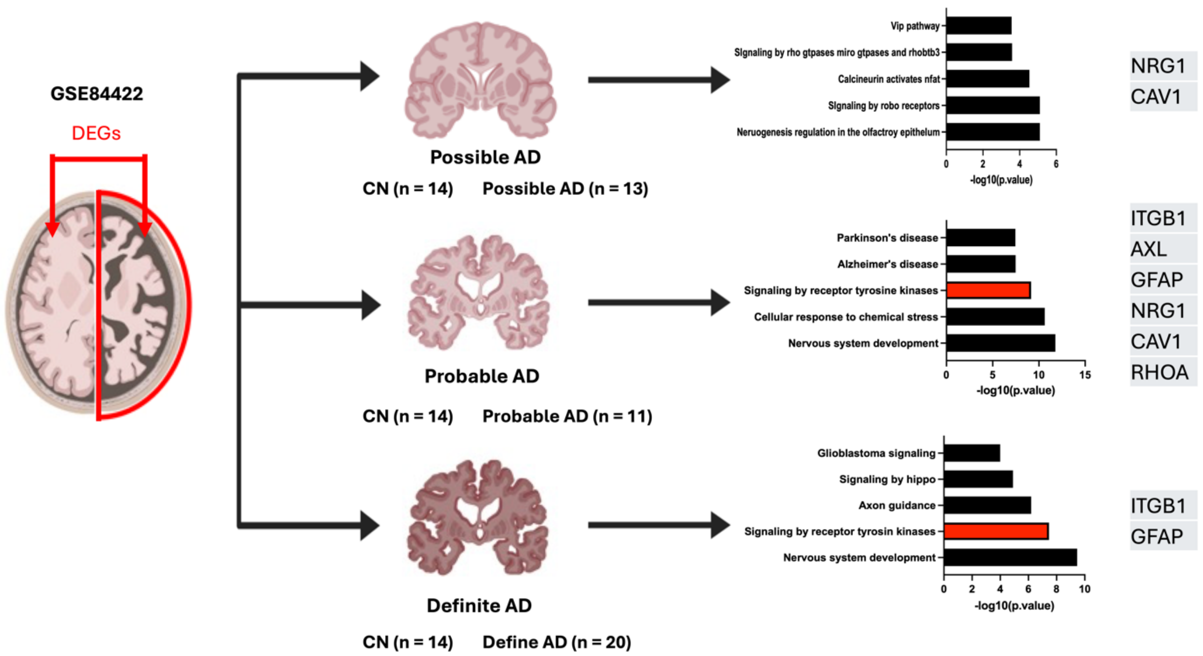

DEGs were identified from the GSE84422 dataset and analyzed across different AD stages: Possible AD, Probable AD, and Definite AD. CN and AD cases are shown for each stage. The bar graphs represent enriched pathways and associated oDEG identified at each stage. Possible AD shows limited RTK activation, while probable and definite AD stages exhibit progressively increased RTK pathway involvement. Key genes such as *ITGB1*, *AXL*, *GFAP*, *NRG1*, *CAV1*, and *RHOA* were identified at different stages, suggesting a progressive emergence of RTK-related dysregulation during AD pathogenesis. In possible AD, no significant activation of the RTK pathway was observed. However, *NRG1* and *CAV1* were identified within the DAG set. In probable AD, RTK pathway activation was detected, with multiple DAGs, including *ITGB1*, *AXL*, *GFAP*, *NRG1*, *CAV1* and *RHOA*, showing differential expression. By the definite AD stage, RTK pathway activation persisted, with *ITGB1* and *GFAP* demonstrating distinct expression changes.

Supplementary Fig. 3. Western blot analysis of Aβ aggregation states

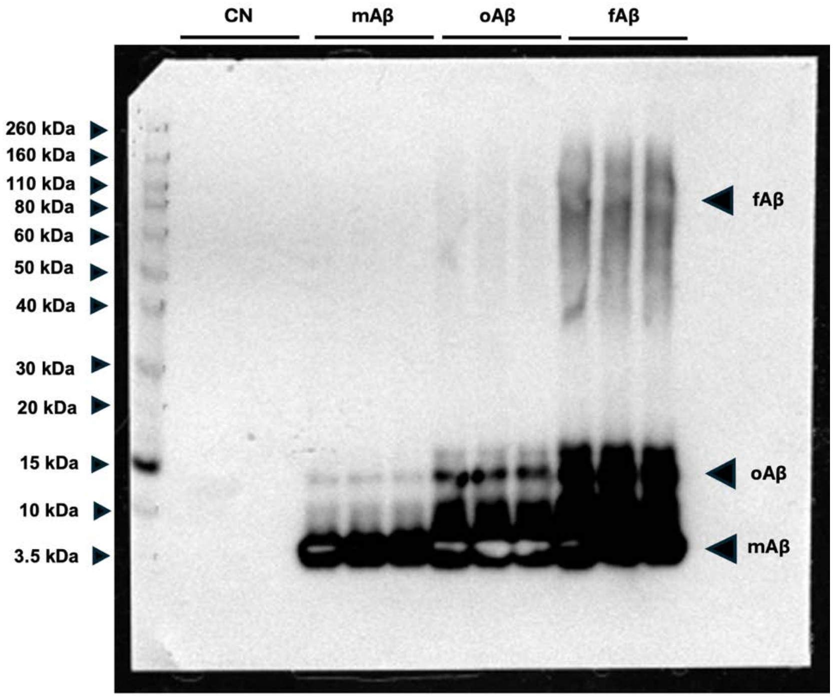

Supplementary Fig. 4. Beta-amyloid and tau secretion from brain organoids.

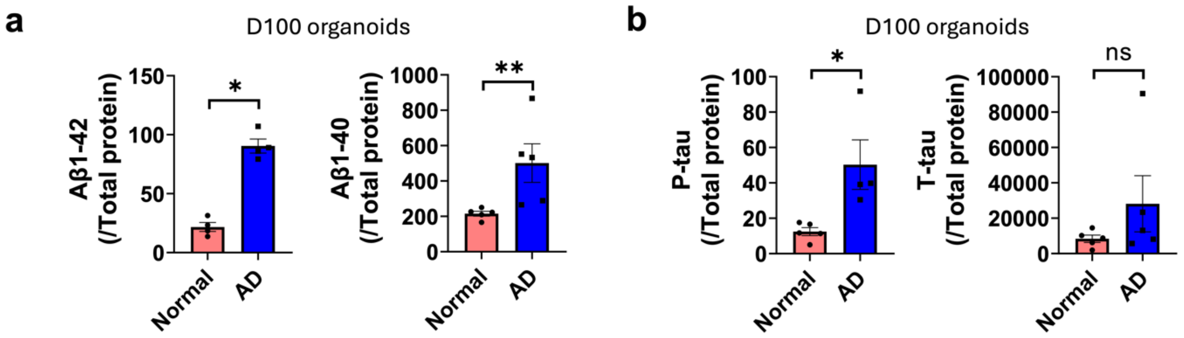

c

iPSC line	Source	Status	Age	Sex
Line 1	PBMC	AD	75	F
Line 2	PBMC	AD	76	F
Line 3	PBMC	AD	76	F
Line 4	PBMC	AD	73	F
Line 5	PBMC	AD	70	F
Line 6	PBMC	Normal	58	M
Line 7	PBMC	Normal	61	M
Line 8	PBMC	Normal	74	M
Line 9	PBMC	Normal	76	F
Line 10	PBMC	Normal	76	F

(a) Comparison of the secretion levels of beta-amyloid 1-42 and 1-40 between organoids from cognitively normal (CN) vs organoids from AD patients. *$p < 0.05$ and **$p < 0.01$ by Mann-Whitney test. **(b)** Comparison of the secretion levels of total tau (t-tau) and phosphorylated tau (p-tau) between organoids from cognitively normal (CN) vs organoids from AD patients. *$p < 0.05$ and **$p < 0.01$ by Mann-Whitney test. ns, no significance. **(c)** Information on iPSCs used for brain organoid generation.

Supplementary Fig. 5. Comparison between tendency of six DAGs from brain organoid-based RNA sequencing data of our group (PRJNA678865) and that of six DAGs from human brain transcriptome (GSE84422 and GSE109887).

	RNA seq data				
**Gene**	***P*-value**	**Log_2FC**	**U/D**	**Public DB U/D**	**Up/down is matched? (vs. Public DB)**
AXL	*0.053	1.222	U	U	Yes
ITGB1	*0.065	0.759	U	U	Yes
GFAP	0.929	-0.107	D	U	No
RHOA	0.574	0.169	U	U	Yes
NRG1	0.271	-0.667	D	D	Yes
CAV1	0.359	1.069	U	U	Yes

*DEG: $P < 0.1$ & $\log_2|FC|>1$

DAG, disease-assocated gene; FC, fold change; DEG, differentially expressed genes; U/D, up-regulated or down-regulated; DB, database.

Supplementary Fig. 6. Protein-protein interaction analysis of AXL and ITGB1 across RTK downstream pathways.

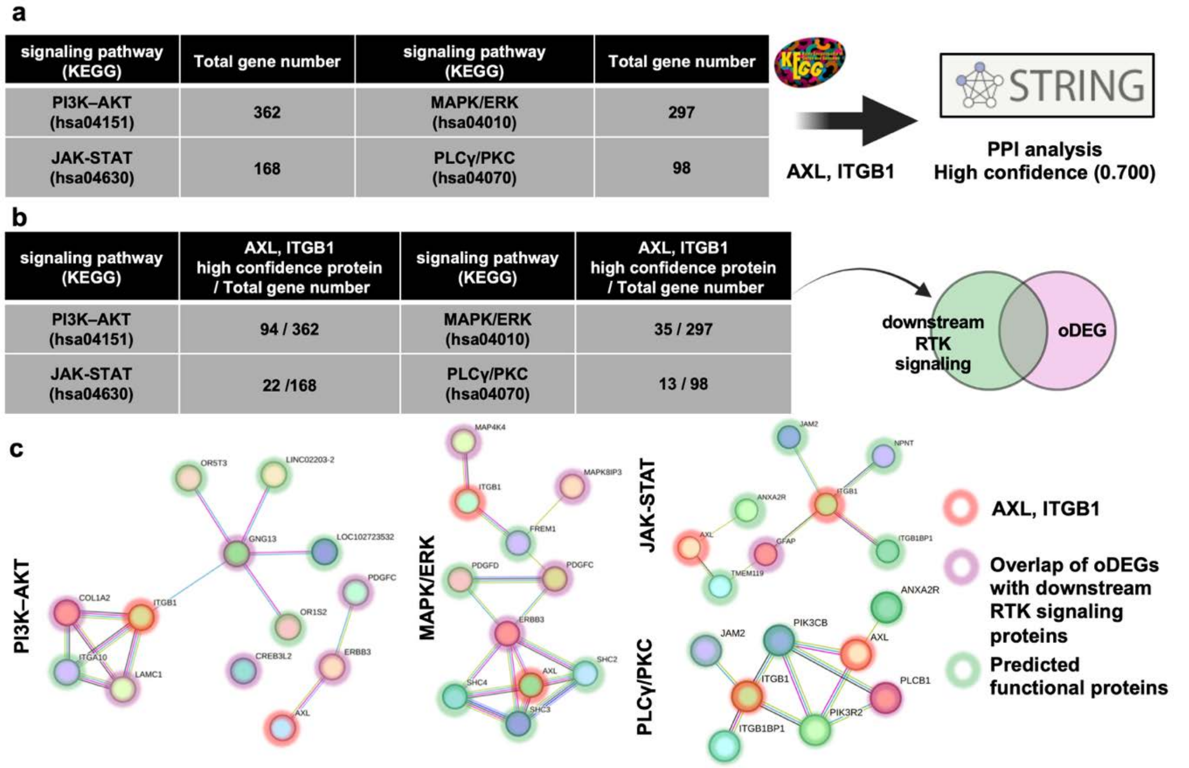

(a) High-confidence (score ≥ 0.7) protein–protein interaction (PPI) networks centered on AXL and ITGB1 within KEGG-annotated pathways: PI3K–AKT (hsa04151), MAPK/ERK (hsa04010), JAK/STAT (hsa04630), and PLCγ/PKC (hsa04070). **(b)** Proteins interacting with AXL and ITGB1 overlapped with the oDEG list in each of the PI3K–AKT, MAPK/ERK, JAK/STAT, and PLCγ/PKC pathways. **(c)** PPI analysis to AXL and ITGB1 across RTK downstream pathways. Red circles represent AXL or ITGB1, purple circles represent overlapping between oDEGs and downstream RTK signaling proteins and green circles predicted functional proteins.

Supplementary Fig. 7. Graphical summary of this study.

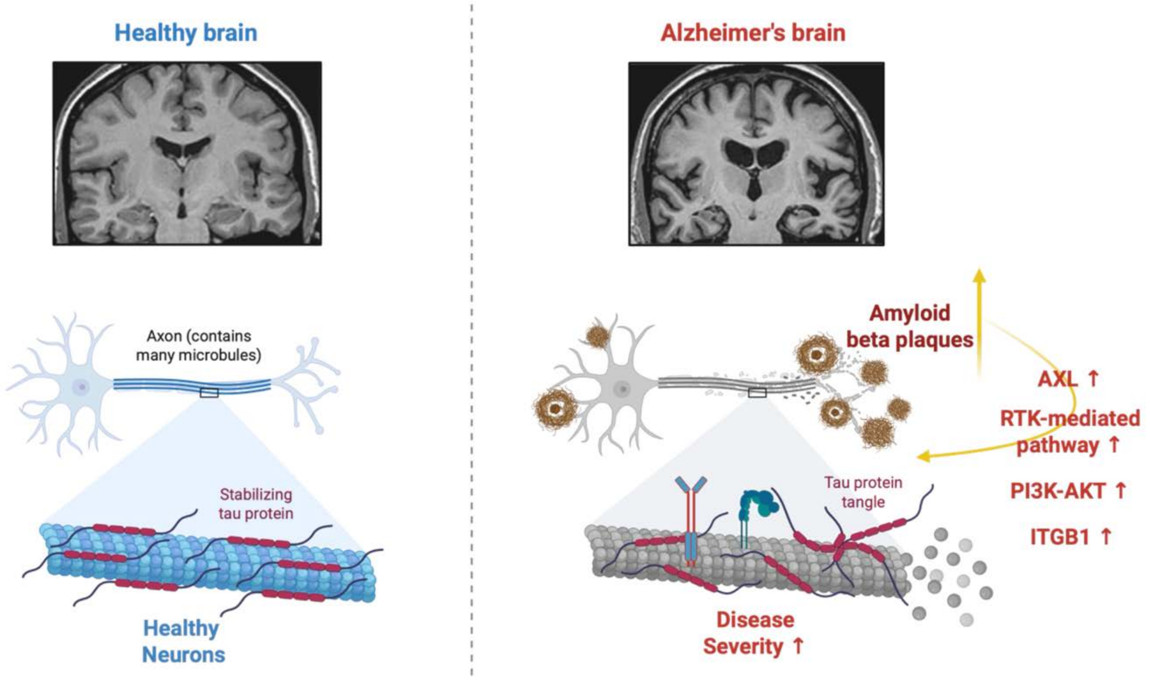

Human brain transcriptome, human brain organoid transcriptome, and bioinformatical/experimental validations with primary neurons revealed possible contributions of RTK pathways to AD pathogenesis. The AXL RTK, ITGB1, and PI3K-AKT pathways were selected as main contributors for AD pathogenesis.

RTK, receptor tyrosine kinase

Supplementary Table 1. Primer list.

**Primer list**			
**Genes**	**RefSeq ID**	**Forward**	**Reverse**
AXL	NM_021913	GTTTGGAGCTGTGATGGAAGGC	CGCTTCACTCAGGAAATCCTCC
CAV1	NM_001753	CCAAGGAGATCGACCTGGTCAA	GCCGTCAAAACTGTGTGTCCCT
GAPDH	NM_002046	GTCTCCTCTGACTTCAACAGCG	ACCACCCTGTTGCTGTAGCCAA
GFAP	NM_002055.5	CTGGAGAGGAAGATTGAGTCGC	ACGTCAAGCTCCACATGGACCT
ITGB1	NM_033667	GGATTCTCCAGAAGGTGGTTTCG	TGCCACCAAGTTTCCCATCTCC
NRG1	NM_013956	GATTCCTACCGAGACTCTCCTC	TGGAAGGCATGGACACCGTCAT
RHOA	NM_001664	TCTGTCCCAACGTGCCCATCAT	CTGCCTTCTTCAGGTTTCACCG
